# Osseointegration of threaded acetabular cups - radiological and histological evaluation after total hip arthroplasty

**DOI:** 10.1007/s00264-025-06687-x

**Published:** 2025-11-04

**Authors:** Elisabeth M. Mandler, Peter Lorenz, Stephanie Huber, Jochen G. Hofstaetter, Lena Hirtler, Gilbert M. Schwarz

**Affiliations:** 1 Center for Anatomy and Cell Biology, Medical University of Vienna, Vienna, Austria; 2 Michael Ogon Laboratory for Orthopaedic Research, Orthopaedic Hospital Speising, Vienna, Austria; 3 2nd Department, Orthopaedic Hospital Speising, Vienna, Austria; 4 Michael Ogon Laboratory for Orthopaedic Research, Orthopaedic Hospital Speising, Vienna, Austria; 5 Department for Orthopaedic and Trauma Surgery, Medical University of Vienna, Vienna, Austria; 6 Center for Anatomy and Cell Biology, Medical University of Vienna, Vienna, Austria

**Keywords:** Osseointegration, Cementless threaded acetabular cups, Total hip arthroplasty

## Abstract

**Purpose:**

Aseptic loosening of the acetabular cup component is the primary cause for complex revision surgery following total hip arthroplasty. However, the extent to which the different zones of the prosthetic contribute to successful osseointegration and how reliable this integration can be assessed using conventional radiographs remain unclear. The aim of the study was to evaluate the osseointegration of cementless threaded acetabular cups through a combination of radiological analysis and histological validation.

**Methods:**

Eight hemipelves of body donors with cementless threaded acetabular cups were included in this study. Conventional radiographs were used to assess the specimens for existing radiolucent lines, periprosthetic osteolysis, or fractures. For histological analysis, thin sections of the acetabular cup were examined for the presence of a periprosthetic membrane, particle debris or inflammatory cells. The areas of visible contact were identified and the bone-to-implant contact (BIC) was calculated.

**Results:**

Radiographic analysis revealed no signs of insufficient osseointegration, osteolytic lesions, or periprosthetic loosening, in any of the specimens. Histological examination showed an average osseointegration rate of 41.84%. Bone-to-implant contact analysis showed no significant differences between different sectors or zones, or between conical and bi-conical acetabular cups.

**Conclusion:**

This study highlights successful osseointegration of cementless threaded acetabular cups, with a mean survival of 18.2 years. Radiological imaging aligned closely to histological finding, confirming implant stability and long-term clinical effectiveness.

**Supplementary Information:**

The online version contains supplementary material available at 10.1007/s00264-025-06687-x.

## Introduction

In recent decades, rising life expectancy has led to a surge in degenerative joint diseases especially in the large weightbearing joints such as hip and knee joints [1]. In this, specifically osteoarthritis affects many elderly individuals, contributing to the high demand for hip replacement surgeries in particular [2]. Even though the total hip arthroplasty (THA) has become one of the most common operations in the field of orthopaedics, complications can arise, often necessitating revision surgeries [3, 4]. Leading causes for these surgical revision include aseptic loosening, periprosthetic infection (i.e. septic loosening) and dislocation [5, 6].

Cementless threaded acetabular cups were developed as response to concerns about the so called “cement disease”, with fixation achieved through direct structural connection between the implant and surrounding bone [7]. This refers to a process called osseointegration and is achieved through periprosthetic osteogenesis and bone remodeling. Osseous integration is crucial for maintaining implant stability, which directly contributes to the implant’s long term survival [8–10]. Should its osseous integration be incomplete, a layer of connective tissue forms around the implant referred to as the periprosthetic membrane. This membrane can be classified into one of four types, each characterized by a specific histological morphology and underlying etiology. While periprosthetic membranes can also be present in stable endoprostheses, they are typically thinner than in unstable implants [11].

Conical threaded acetabular cups, along with their successors, the bi-conical cups-designed to reduce bone loss and enhance tilt stability - were gradually replaced in recent years by spherical press-fit acetabular cups. While press-fit cups were initially introduced to minimize the risk of osteolysis around the threads, newer generations of threaded cups showed similar long-term survival rates [12]. Nevertheless aseptic loosening remains the number one reason for implant failure irrespective of the implant type [13]. Two mechanisms lead to aseptic loosing: (1) material wear particles stimulating macrophages, leading to osteolysis and (2) primary implant instability [14].

The extent to which the different sides of a threaded acetabular cup contribute to successful osseointegration and how reliable this integration can be assessed using conventional x-ray still remains unclear. Because these implants were widely used in recent decades, there are still many patients with threaded acetabular cups in situ today, who may present with periprosthetic fractures or hip pain. The study therefore aims to evaluate the osseointegration of cementless threaded acetabular cups through radiological analysis and histological validation.

## Materials and methods

All specimens in this anatomical study originated from the Medical Bio-/Implantbank Vienna and were obtained from individuals, who, during their lifetime, voluntarily donated themselves to (the Division of Anatomy, Center for Anatomy and Cell Biology of the Medical University of Vienna) for medical teaching and research purposes. Approval from the ethics committee of the (Medical University of Vienna) was obtained (EK. Nr.: 1102/2022). Inclusion criteria consisted of all specimens with cementless threaded acetabular cups as well as a donor age between 18 and 99, and prosthesis age between six months and 35 years. Exclusion criteria included a prosthesis age of less than six months, due to the occurrence of unspecific postoperative inflammation in such cases. According to these criteria, eight specimens were included in this study.

The identification of donors with cementless threaded acetabular cups was achieved using a mobile x-ray machine and through the use of data collected from patient medical records. After identification, the pelvic bones with included implant were collected and the pelvic ring bisected. The resulting hemipelves were vacuum packed and stored at -20 °C until evaluation. Donor age, sex, year of decease, cause of death, implant side, size, age and implant material as well as applied surgical technique were determined from the patients’ histories.

The hemipelves were radiologically examined using a standardized examination box and an x-ray calibration sphere. The box was filled with water to simulate soft tissue [15]. Conventional radiographs from anterior-posterior (AP) and acetabular overview positions were performed on each specimen in order to detect existing radiolucent lines, periprosthetic osteolysis or fractures.

**Fig. 1:**
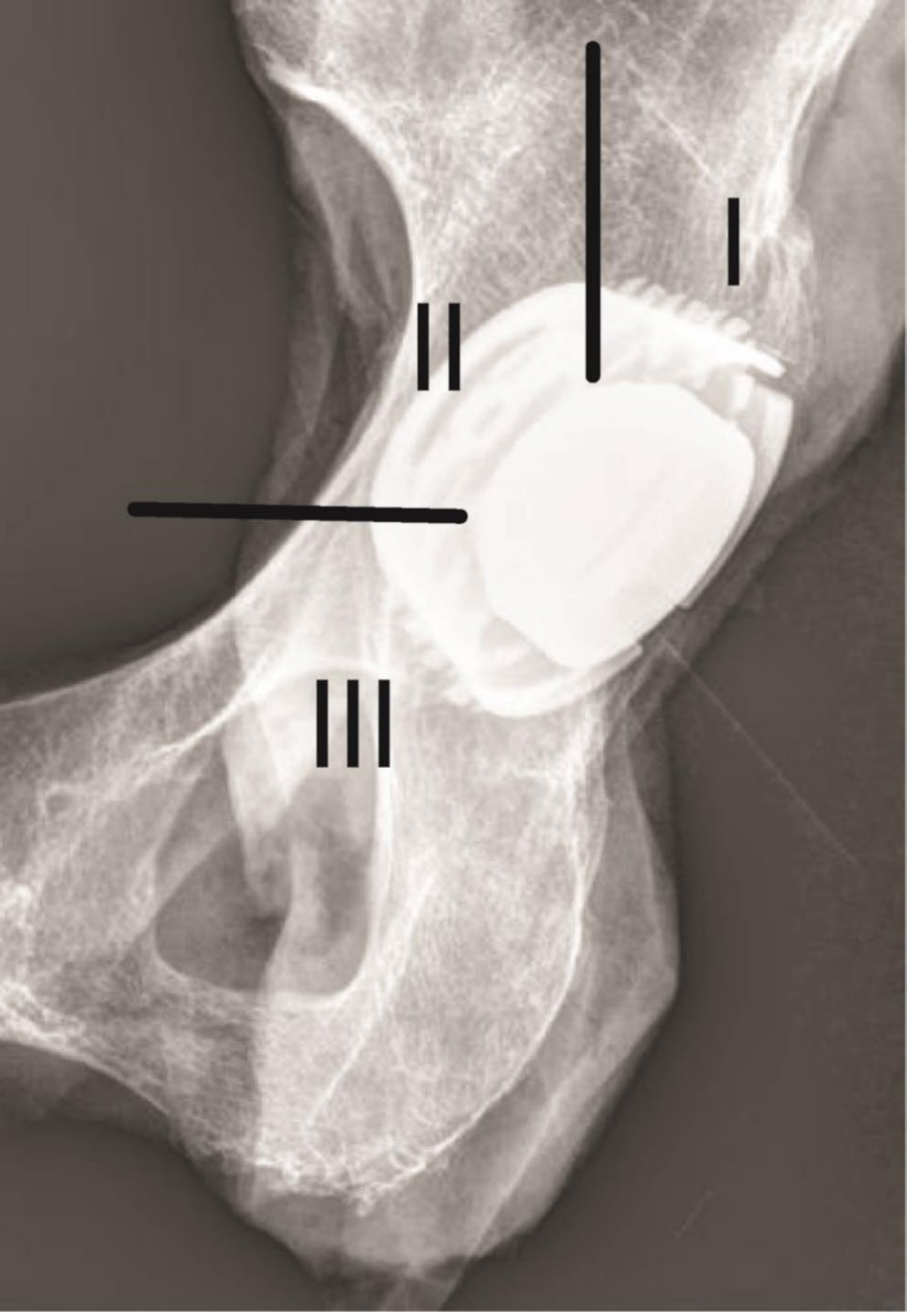
DeLee and Charnley zones I, II, III (Figure created according to Miller [17])

The AP radiographs were divided in three zones described by DeLee and Charnley [16], (Fig. 1) with the aim of facilitating a more detailed description of periprosthetic loosening. Lucent lines over 2 mm surrounding the acetabular cup were classified as a sign of periprosthetic loosening.

**Fig. 2:**
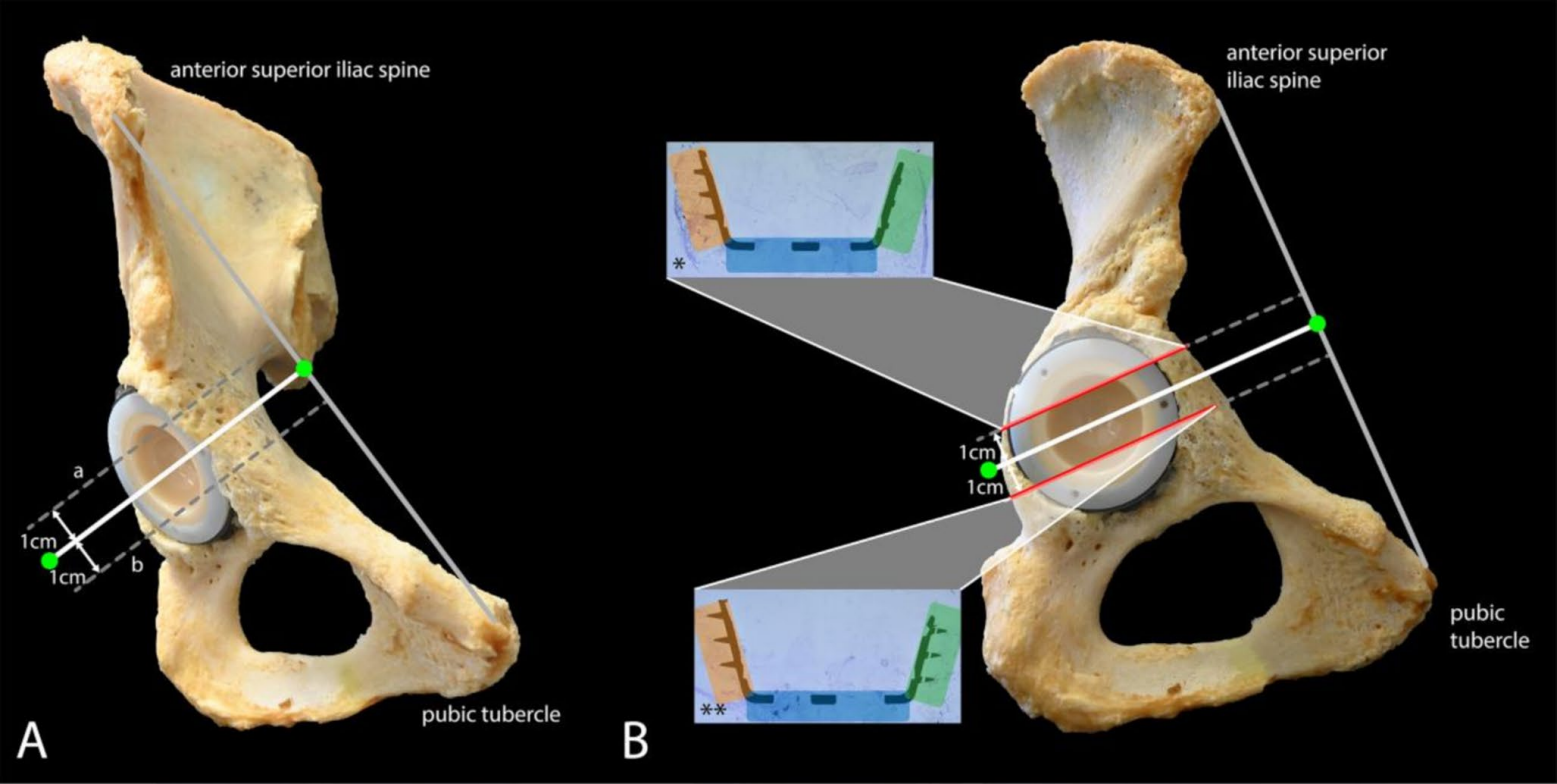
Hemipelvis with schematic incision. **A**) The initial incision was made through the center of the acetabular cup (white line), with two additional parallel incisions at 1 cm intervals, creating a superolateral (a) and inferomedial (b) section. **B**) Thin sections through the * superolateral (zone 1) and ** inferomedial (zone 3) area of the implant, corresponding to the DeLee and Charnley zones (1976). The implant margin highlighted in green represents zone 1AS, the red-highlighted margin represents zone 3PI and the blue-highlighted margin represents zone 2B

**Fig. 3:**
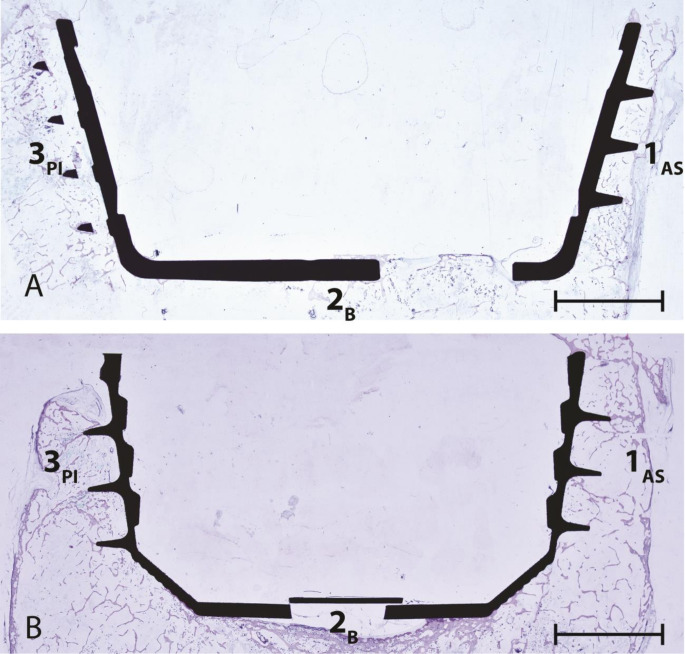
Thin section subdivision into zones: zone 1 corresponds to the anterior-superior, zone 2 to the base and zone 3 to the posterior-inferior part of the acetabulum, (**a**) bi-conical acetabular cup (**b**) conical acetabular cup. Scale bar signifies 10 mm

For the histological evaluation using thin-ground sections, one inferomedial section through DeLee and Charnley zone 1 and one superolateral section through zone 3 were obtained from each acetabulum using a diamond band saw (EXAKT^®^ 312 pathology saw) (Fig. 2A). These sections were selected in order to illustrate various implant anchorage points of the acetabular cup and to capture the maximal curvature of the implant. Zone 2 of DeLee and Charnley is defined as the base of the implant, this was thus included in each histological slide. For a more detailed analysis, each histological section through either Zone 1 or Zone 3 was subdivided into three sectors (Fig. 2B). Sector 1_AS_ represented the anterior-superior portion of the acetabular cup, sector 2_B_ (equal to DeLee and Charnley zone 2) was the base and sector 3_PI_ the posterior-inferior part of the acetabular cup (Fig. 3A). The base of bi-conically formed acetabular cups had a central and an oblique surface, together forming section 2_B_ (Fig. 3B). Each sample was marked to ensure accurate identification and orientation.

Fixation of the sections was achieved by Schaffers-solution (formaldehyde and ethanol 1:1), and dehydration using a graded alcohol sequence. This was followed by the embedding of each specimen in a methyl-methacrylate resin mixture. Following a series of grinding and polishing to produce histological slides, they were stained with a Giemsa solution. Two out of the sixteen thin ground sections had to be excluded from histological evaluation due to technical issues during the embedding or grinding process.

The presence of a periprosthetic membrane, particle debris or inflammatory cells were histologically evaluated and a section overview photographed using a commercially available camera (Nikon^®^ D810, Tokyo; Japan). The interpretation of the periprosthetic membrane was based on the consensus classification of Morawietz et al. [11]. Areas of visible bone contact in the images were measured using the image analysis program DotDotGoose (https://biodiversityinformatics.amnh.org/open_source/dotdotgoose/ American museum of Natural History, New York, USA).

In DotDotGoose, the following classes were defined: Bone Contact Sector 1_AS_, 2_B_, 3_PI_, and No-Bone Contact Sector 1_AS_, 2_B_, 3_PI_. Measurements were taken using a point radius of 10 and a grid size of 35. To determine the area of bone-to-implant contact (BIC), each box of the grid in which both bone tissue and the prosthetic were present was marked with four dots. Green dots represented contact between the bone and implant, while red indicated the absence of contact. Then, the BIC was calculated by using the following formula.

$$\:BIC=\:\frac{Bone\:Contact\:Points\:Side\:\left(x\right)}{(Bone\:Contact\:Points\:\left(x\right)+No\:Bone\:Contact\:Points\:\left(x\right))}$$

## Statistics

For all metric data, mean values and standard deviation were calculated using SPSS Inc. (Version 16.0, Chicago, USA). Using a Shapiro-Wilk-Test, BIC values for each of the three sides and the overall BIC were tested for normal distribution. The BIC values of the inferomedial and superolateral section of sector 1_AS_, sector 2_B_ and of the overall BIC as well as the superolateral section of sector 3_PI_ all showed normal distribution. Only the BIC value of the inferomedial section of sector 3_PI_ did not show normal distribution (*p* < 0,05). Given that all except one variable showed equal distribution, a paired t-Test was used to compare the values. *P*-values were corrected through the use of the Bonferroni-Holm method. Corrected *P*-values of < 0.05 were seen as statistically significant.

## Results

This study included eight hemipelves with cementless threaded acetabular cups. The body donors had an average age of 85.6 years, with an equal distribution of male and female specimens. The average implant life was 18.2 years. Among the implants, three were conical and five bi-conical acetabular cups. The demographic data is presented in Table 1.

**Table 1:** Dempgraphical overview

Specimen number	Age (years)	Sex	Side	Implant survival (years)	Implant type
1	76	Female	left	26.9	bi-conical
2	79	Male	right	> 6 months	bi-conical
3	92	Female	right	> 6 months	bi-conical
4	93	Male	left	17.0	bi-conical
5	96	Female	left	24.7	bi-conical
6	74	Male	left	5.1	conical
7	82	Female	left	16.6	conical
8	93	Male	right	19.0	conical

### Descriptive analysis of radiographs

The radiographs showed no indication of insufficient osseointegration in either the anterior-posterior or the acetabular overview position. None of the eight specimens displayed signs of osteolytic lesions or periprosthetic loosening, which would be indicated through visible radiolucent lines exceeding 2 mm [17]. The radiographic images displayed the shape of each prosthetic, showing three conical and five bi-conical acetabular cups.

### Descriptive analysis of histological thin-ground sections

**Fig. 4:**
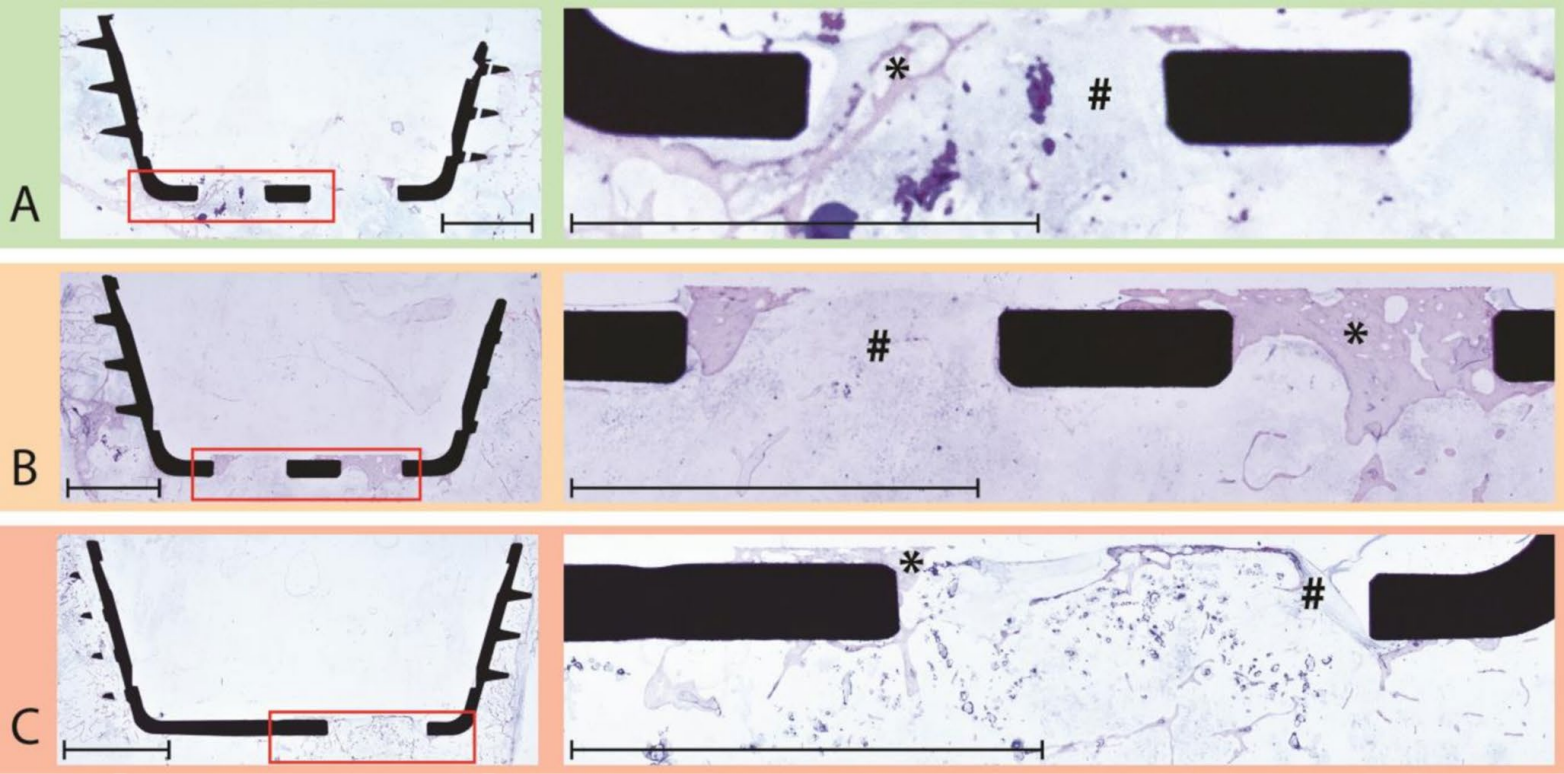
Example of three base surfaces with ingrown bone (*) and connective tissue (#). Scale bar signifies 10 mm

**Fig. 5:**
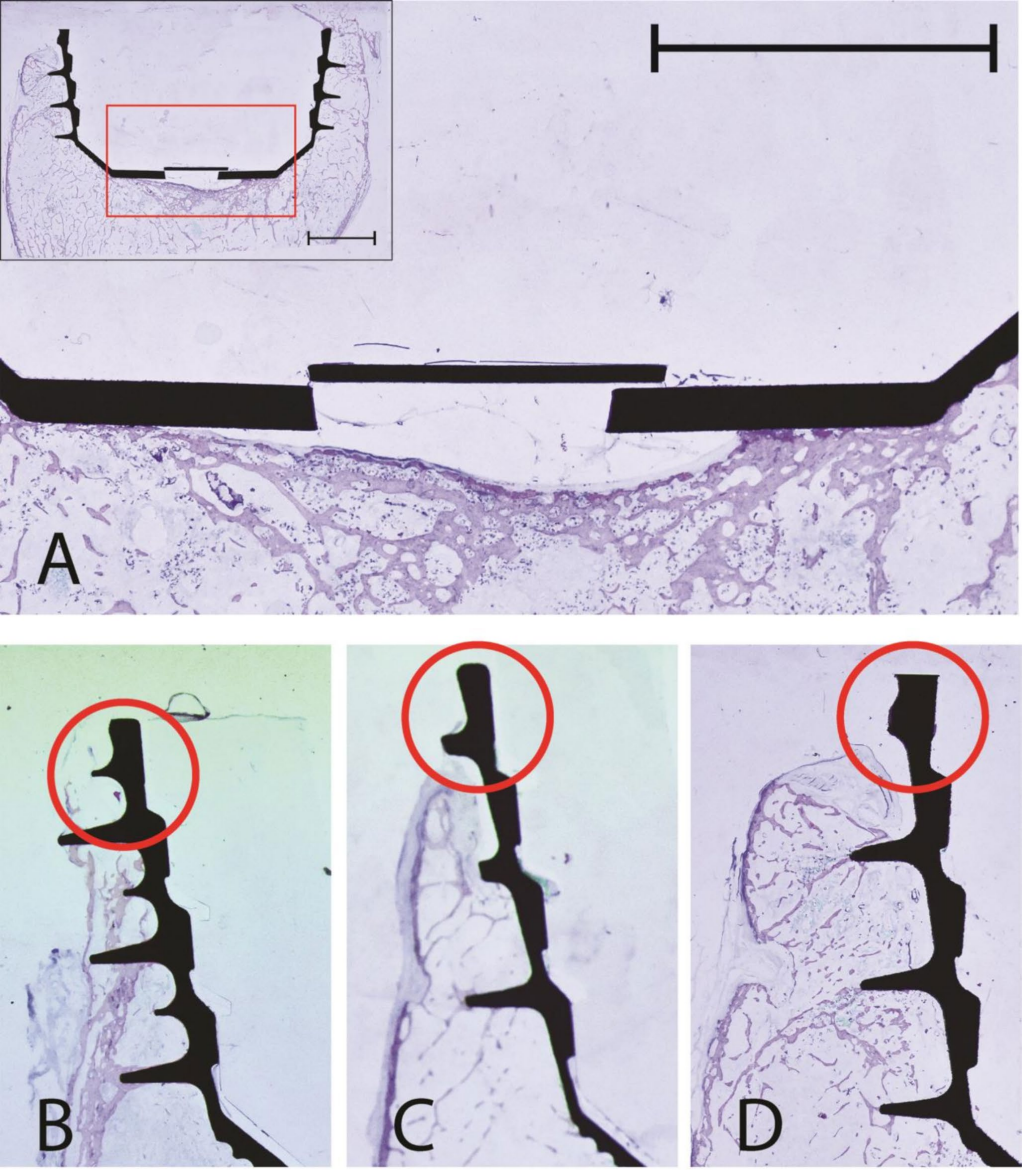
**A**: Cavity between the base of the prosthesis and the bone. Scale bar signifies 10 mm. **B**-**D**: inferioposterior acetabular rim without bone contact in thin sections of three different specimens (marked using red circles)

A clear distinction between the prosthetic material and surrounding bone tissue was evident in all fourteen thin-ground sections. The cortical bone showed multiple osteons with their respective Haversian-canals, making it easily distinguishable from the trabecular structure of the spongy bone. A periprosthetic membrane, was observed in seven thin-ground sections, belonging to four specimens, consistently located in sector 2_B_. Inflammatory cells were not identified in any of the specimens. In all thin sections, the outer edge of the prosthetic consistently made contact with the bone in sector 1_AS_ and 3_PI_. Three specimens exhibited an opening in sector 2_B_ of the implant, in which either bone or connective tissue were present in all corresponding thin sections (Fig. 4). In one specimen, an osseous cavity formed between the bone and the base of the prosthetic (sector 2_B_), visible only in the inferomedial section. Additionally, a periprosthetic membrane was clearly present in this section (Fig. 5A). Notably, in three thin sections, the dorsal-posterior margin showed no contact with either bone or connective tissue (Fig. 5B-D).

### Statistical analysis of bone-to-implant contact (BIC) in histological sections

All data collected for the BICs of each sector (1_AS_, 2_B_, 3_PI_) and section (inferomedial and superolateral), are displayed in Supplementary Table 1 in the appendix. Calculated *p*-values from paired T-Tests are presented in Supplementary Table 2.

Comparing the mean BIC values between the inferomedial and superolateral section of each specimen revealed quantitative differences, with the inferomedial section showing higher BIC values. Similar results were achieved when comparing the mean BIC values of the individual sectors. However, no statistically significant difference was found. When comparing the base (sector 2_B_) with the combined lateral surfaces (sector 1_AS_ and 3_PI_), no significant difference in osseointegration was observed (*p* = 0.425, after Bonferroni correction: *p* = 0.85). Analysis of conical (mean value: 0.44) and bi-conical (mean value 0.41) acetabular cups revealed no significant difference in osseointegration (*p* = 0.304, after Bonferroni correction: *p* > 0.999). The rate of osseointegration, defined as the implant surface with direct bone contact, was 41,84%, as determined by the average BIC across all sections.

## Discussion

This study assessed osseointegration in long-term implanted cementless threaded acetabular cups as a key indicator of implant stability, measured by bone-to-implant contact (BIC), which quantifies the direct connection between the prosthetic implant and surrounding bone tissue. Histological sections and corresponding radiographs of anatomical specimens were analyzed to assess direct bone-implant connection. The average BIC of 41.89% found in this study indicates a substantial amount of bone in direct contact with the implants, thereby showing successful osseointegration, without osteolysis around the threads. This finding is further supported by the average implant life of 18.2 years.

Comparing these results with the work of Bloebaum et al. [18], which analyzed seven press-fit acetabular cups microradiographically, a notable contrast emerges. They calculated the appositional bone index, similar to the BIC, to quantify bone contact with the implant surface. Bloebaum et al. reported an average appositional bone index of 84% ± 9%. Their results indicate a high degree of bone integration, showing a substantial difference in BIC compared to our findings. These discrepancies may be attributed to differences in implant designs as we investigated threaded acetabular components.

Tonino et al. [19] also evaluated spherical press-fit acetabular cups of six post-mortem hips with hydroxyapatite coating and twelve screw holes to assess bone-to-implant contact. Their findings revealed an average BIC of 36.5% ± 13.5%, which is substantially lower than the results of Bloebaum, although similar cup designs were investigated. BIC values in our study were in between those of Bloebaum and Tonino. Additionally, we observed comparable results regarding the absence of statistically significant differences in BIC concerning the DeLee and Charnley zones.

We compared the osseointegration of conically shaped and bi-conically shaped threaded acetabular cups to investigate whether implant geometry influenced bone-implant contact. This comparison revealed no statistically significant difference in osseointegration (*p* = 0.304, after B-H correction: *p* > 0.999) between conical (mean: 44%) and biconical (mean: 41%) cups. These results suggest that implant geometry may not significantly impact bone-implant contact. Nevertheless, geometric differences could still influence other aspects, such as initial stability, which was not assessed in this study.

An extensive search through the literature found no evidence of varying degrees of osseointegration between different sections of the same specimen. This suggests that the differences in BIC values observed between the inferomedial and superolateral sections in our study are not significant.

A periprosthetic membrane was found in seven histological sections from four specimens; however, there was no indication of loosening, inflammation or osteolysis, confirming implant stability. Radiographic analysis showed no signs of loosening, which was consistent with histological findings. Based on these results, it can be concluded that all examined acetabular implants were stably anchored.

In the histological analysis of press-fit acetabular cups by Bloebaum et al. [18], polyethylene and metal abrasion particles were observed along six of ten screws. In contrast, our study involving threaded acetabular cups, identified no such particles. Similarly, no macrophages or giant cells were detected, whereas Bloebaum reported a few, though they showed no signs of active inflammation.

Although threaded acetabular cups have been largely replaced by press-fit cups in most countries, recent studies have shown that the newer threaded cups demonstrate similar long-term results [12]. Despite their comparable performance and longer existence, these newer threaded cups have never been histologically examined postmortem with the exception of one case report from 1988 [20]. Because these implants were widely used in recent decades, there are still many patients with threaded acetabular cups in situ today, who may present with periprosthetic fractures or hip pain. The current research highlights that, threaded acetabular cups can still offer successful osteointegration without lysis and demonstrate long-term survival.

### Limitations

The primary limitation of this study was the availability of long-term implanted prostheses of body donor specimens, resulting in a sample size of only eight specimens. While this sample size is adequate for an anatomical study, it may impact the statistical significance of findings. In this study, specimens were x-rayed because radiographs are commonly used clinically for postoperative follow-up and complication assessment [21]. While CT scans provide cross-sectional data for more detailed analysis, they were avoided due to metal-induced streak artifacts, which can distort the bone-implant interface [15]. Given these shortcomings, radiographs were the preferred imaging method for reliable evaluation. An additional limitation was that two out of the sixteen thin sections could not be used for histological evaluation due to issues during the grinding process making it impossible to calculate the total BIC value and that of each side. Although prosthetic loosening is typically diagnosed on clinically presented symptoms, which were not considered in this study, we believe this research offers valuable insights into osseointegration of long-term threaded acetabular cup implants and underlines the relevance of the available data.

## Conclusion

This study demonstrates the successful osseointegration of cementless threaded acetabular cups, with a mean survival rate of 18.2 years. Bone-to-implant contact was consistently observed, along with a visible periprosthetic membrane in four of the eight specimens. No significant differences in bone-to-implant contact were observed between sections or sectors of the cup. Radiological assessment confirmed the histological findings, showing no evidence of implant loosening, or osteolysis around the threads and highlighting a strong radiological-histological correlation. These results indicate that the acetabular cups exhibit stable osseointegration, supporting their long-term clinical effectiveness.

## Supplementary Information

Below is the link to the electronic supplementary material.

Supplementary Material 1

## Data Availability

No datasets were generated or analysed during the current study.
