# Supplementary material for: Osseointegration of threaded acetabular cups - radiological and histological evaluation after total hip arthroplasty
Source: Int Orthop. 2025 Nov 4;50(1):101–9. doi: 10.1007/s00264-025-06687-x (PMC12880993; doi:10.1007/s00264-025-06687-x)
Supplement: Supplementary file 1 — Supplementary Material 1 [file 264_2025_6687_MOESM1_ESM.docx]

**Supplemetary files**

| Sector | Section | BIC-Mean Values | Standard Deviation |
| --- | --- | --- | --- |
| 1_AS_ | inferomedial | 0.475 | 0.117 |
| 1_AS_ | superolateral | 0.372 | 0.134 |
| 2_B_ | inferomedial | 0.466 | 0.239 |
| 2_B_ | superolateral | 0.407 | 0.145 |
| 3_PI_ | inferomedial | 0.416 | 0.130 |
| 3_PI_ | superolateral | 0.358 | 0.125 |
| Total | inferomedial | 0.452 | 0.144 |
| Total | superolateral | 0.384 | 0.111 |

Supplementary Table 1 Mean values and standard deviation of the BIC values

| BIC Measuring Point | p-Value | Bonferroni correction |
| --- | --- | --- |
| BIC sector 1_AS_ – BIC sector 2_B_ | 0.771 | > 0.999 |
| BIC sector 1_AS_ – BIC sector 3_PI_ | 0.423 | > 0.999 |
| BIC sector 2_B_ – BIC sector 3_PI_ | 0.329 | > 0.999 |
| BIC sector 1_AS_ (inferomedial) – BIC sector 1_AS_ (superolateral) | 0.295 | > 0.999 |
| BIC sector 2_B_ (inferomedial) – BIC sector 2_B_ (superolateral) | 0.584 | > 0.999 |
| BIC sector 3_PI_ (inferomedial) – BIC sector 3_PI_ (superolateral) | 0.8 | > 0.999 |
| BIC total (inferomedial) – BIC total (superolateral) | 0.768 | > 0.999 |

Supplementary Table 2 p-values of the BIC before and after Bonferroni correction
